# The survival rate of transcrestal sinus floor elevation combined with short implants: a systematic review and meta-analysis of observational studies

**DOI:** 10.1186/s40729-021-00325-y

**Published:** 2021-05-20

**Authors:** Zhe-Zhen Lin, Yan-Qing Jiao, Zhang-Yan Ye, Ge-Ge Wang, Xi Ding

**Affiliations:** Department of Stomatology, The First Affiliated Hospital of Wenzhou Medical University, Nanbaixiang Ouhai District, Wenzhou, 325000 Zhejiang People’s Republic of China

**Keywords:** Short implant, Sinus floor augmentation, Dental implant, Meta-analysis

## Abstract

**Background:**

Currently, insufficient bone volume always occurs in the posterior maxilla which makes implantation difficult. Short implants combined with transcrestal sinus floor elevation (TSFE) may be an option to address insufficient bone volume.

**Purpose:**

The clinical performance of short implants combined with TSFE was compared with that of conventional implants combined with TSFE according to the survival rate.

**Method:**

In this systematic review and meta-analysis, we followed the Meta-Analysis of Observational Studies in Epidemiology (MOOSE) guidelines. Articles were identified through PubMed, Embase, the Cochrane Library, and manual searching. Eligibility criteria included clinical human studies. The quality assessment was performed according to the Strengthening the Reporting of Observational Studies in Epidemiology (STROBE) guidelines. The odds ratio (OR) with its confidence interval (CI) was considered the essential outcome for estimating the effect of short implants combined with TSFE.

**Results:**

The registration number is INPLASY202050092. Eleven studies met the inclusion criteria, including 1 cohort study and 10 cross-sectional studies. With respect to the 1-year survival rate, no significant effect was observed between short implants (length ≤ 8 mm) and conventional implants combined with TSFE (*I*^2^=0%, OR=1.04, 95% CI: 0.55-1.96). Similarly, no difference was seen between the two groups regarding the survival rate during the healing period (*I*^2^=10%, OR=0.74, 95% CI: 0.28-1.97) and 3-year loading (OR=1.76, 95% CI: 0.65-4.74).

**Conclusion:**

There was no evidence that the survival rate of short implants combined with TSFE was lower or higher than that of conventional implants combined with TSFE when the residual bone height was poor and the implant protrusion length of short implants was less than or similar to conventional implants. Nevertheless, the results should be interpreted cautiously due to the lack of random controlled trials in our meta-analysis.

**Supplementary Information:**

The online version contains supplementary material available at 10.1186/s40729-021-00325-y.

## Introduction

Currently, dental implants are widely used in dentition defects. However, insufficient bone volume always occurs in the posterior maxilla, which makes implantation difficult. For this reason, several methods have been applied to address this problem, such as short implants, tilted implants, and sinus lift.

Lateral sinus floor elevation, introduced by Boyne and James in 1980 [1], is a conventional method utilized in situations with insufficient bone height. Considering that this method is invasive and time-consuming, a modified technique is required in the oral clinic, and osteotome sinus floor elevation was initially proposed by Tatum and Summers [2, 3], to decrease unnecessary trauma and complications. This technology has been proven to be an excellent choice when the available bone height exceeds 5 mm [4, 5]. Nevertheless, Schneiderian membrane laceration may occur when struck by an osteotome due to its invisibility, but perforation may not result in a significant reduction in survival rates. The perforation site serves as a channel between the maxillary sinus and the surrounding environment, increasing the risk of infection with original sinusitis. Another important intraoperative complication is benign paroxysmal positional vertigo (BPPV), and several investigators have suggested that the frequency of BPPV is approximately 3% [6].

With the development of implant surface modification, short implants are an alternative to sinus floor elevation [7], and they reduce the possibility of complications and simplify implant surgery procedures. In recent years, the definition of short implants has been a controversial topic in academia. Some researchers have maintained that the length of a short implant is less than or equal to 8 mm, while others have insisted that short implants are 6 mm or less [8]. In our research, implants equivalent to 8 mm and below were considered short implants [9–11]. And the implants equivalent to 6 mm and below were extrashort implants [12, 13].

However, when the residual bone height is seriously insufficient, it is not feasible to perform crestal sinus elevation combined with conventional length implants or short implants without sinus floor elevation. At this point, the traditional option is performing external maxillary sinus elevation. To lessen discomfort and trauma, many doctors have adopted simultaneous short implants (length ≤ 8 mm) combined with osteotome sinus floor elevation [14, 15], which is regarded as a reasonable substitute for lateral sinus elevation. The author believes that the combination of crestal sinus floor elevation and short implants is an effective approach.

Several systematic reviews have compared short implants without TSFE with conventional implants plus TSFE, whereas no systematic review has mentioned the clinical outcome of short implants (length ≤ 8 mm) combined with TSFE [7, 16].

The purpose of this study is to review the literature regarding short implants (length ≤ 8 mm) combined with crestal sinus floor elevation. We attempted to compare the survival rate of short implants (length ≤ 8 mm) with that of conventional implants used in crestal floor elevation. We hypothesize that the survival rates of short implants (length ≤ 8 mm) combined with TSFE are similar to those of conventional implants when the implant protrusion length of the short implants is less than or similar to that of conventional implants.

## Materials and methods

This research followed the Meta-Analysis of Observational Studies in Epidemiology (MOOSE) guidelines. The study has been registered in INPLASY, and the number of registrations is INPLASY202050092. Our DOI number is 10.37766/inplasy2020.5.0092. In addition, all the included studies met ethical guidelines and followed the Declaration of Helsinki.

### Search strategy

The majority of articles published between 1994 and 2020 were found in PubMed, EMBASE, and the Cochrane Library in April 2020 [3]. The key items for our search were as follows: (“sinus”) AND (“internal” OR “osteotome” OR “crestal” OR “transcrestal” OR “inlay” OR “indirect”OR “local” OR “localized”) AND (“Dental Implants” OR “Implants, Dental” OR “Dental Implant” OR “Implant, Dental” OR “Dental Prostheses, Surgical” OR “Dental Prosthesis, Surgical” OR “Surgical Dental Prostheses” OR “Surgical Dental Prosthesis” OR “Prostheses, Surgical Dental” OR “Prosthesis, Surgical Dental” OR “implant” OR “oral implant” OR “oral”) [Title/Abstract]. In addition, a few articles were identified through manual searches in different journals: *Periodontology 2000*, *Journal of Dental Research*, *Clinical Implant Dentistry and Related Research*, *Journal of Clinical Periodontology*, *Clinical Oral Implants Research*, *Journal of Dentistry*, *Journal of Periodontology*, *International Journal of Oral Science*, *European Journal of Oral Implantology*, *International Journal of Oral And Maxillofacial Surgery*, *Journal of Periodontal and Implant Science*, *Implant Dentistry*, *British Journal of Oral & Maxillofacial Surgery*, and *Implantologie*.

### Study selection

A large number of duplicate publications were removed by EndNote, and the remaining studies were manually screened. The two authors browsed all the titles and abstracts according to the following criteria:

Inclusion criteria:

Participants (P):

1. The patients were diagnosed with a lack of available bone height in the posterior maxilla, regardless of age, sex, and country (residual bone height < 8 mm).

Intervention (I):

1. Patients underwent osteotome sinus floor elevation, and short implants (length ≤ 8 mm) were simultaneously placed [8].
2. The sample size was at least 15.
3. The residual bone height at the sites with inserted short implants combined with trancrestal sinus floor elevation (TSFE) was 8 mm or less.
4. The implant protrusion length of short implants combined with TSFE was smaller or similar to conventional implants combined with sinus floor elevation.

Comparison (C):

1. Patients underwent sinus floor elevation and immediately received conventional implants (length > 8 mm).
2. There was no limitation on the residual bone height concerning the sites that had conventional implants inserted.

Outcome (O):

1. The principal outcomes were the survival rate of the implants, including the early survival rate and the 1-year, 3-year, and long-term survival rates.
2. Specific details concerning failed implants.

Study design (S):

Randomized controlled trial, observational studies.

Exclusion criteria:

1. Data were repeatedly reported in different articles.
2. The study involved animals, models, or cadavers.
3. Patients only underwent sinus floor elevation or received short implants.
4. The study merely described the postoperative situation.
5. The study was only related to conventional implants (length > 8 mm).
6. The study was a case report or a systematic review.
7. The study was mainly related to orthopedics or cardiology.

Despite these criteria, disagreements occurred in the preliminary screening of abstracts and titles, which compelled us to retain controversial documents for the full-text analysis. Disagreements were assessed by another author to prevent any relevant articles from being included.

**Fig. 1:**
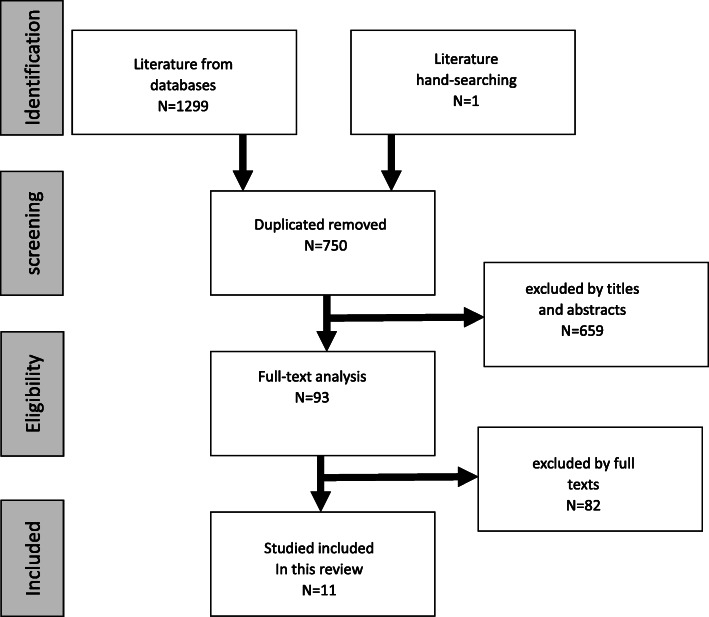
MOOSE (the guidelines of Meta-Analysis of Observational Studies in Epidemiology) program for identification

An overview of the study selection procedure is shown in Fig. 1.

### Quality assessment

The Strengthening the Reporting of Observational Studies in Epidemiology (STROBE) guidelines were applied to the quality assessment [17]. The guidelines consisted of 22 items, according to title and abstract, introduction, method, result, discussion, etc.

### Data extraction

The data was independently extracted by three authors through carefully examining the 11 full texts, and the data included the following variables: (a) first name of the author; (b) date of publication; (c) author’s country; (d) study’s classification; (e) number of patients and implants; (f) patient’s age; (g) healing period; (h) follow-up time; (i) implant system; and (j) primary and secondary outcomes.

### Statistical analysis

Heterogeneity of research was considered based on the Cochran test and *I*^2^ test, which held that the origin of heterogeneity is not accidental but determined by the included articles. Provided that the *I*^2^ value was less than 50%, heterogeneity among the studies was accepted by the researchers [18]. If the *P*-value did not exceed 0.10, these factors were taken into account:

Whether the control group that was selected was appropriate.

Age, sex, and physical condition affected the survival rate.

Whether the available bone height had a significant effect on the survival rate.

The outcomes were analyzed and processed by the methods above, and the random-effects model was adopted if heterogeneity still existed.

For discontinuous variables, the odds ratio (OR), the risk difference (RD), and its 95% confidence interval (CI) were assessed. The results processed by the random-effects model were carefully interpreted, and all the data were analyzed utilizing RevMan. Forest plots of the OR were calculated to demonstrate whether the exposure factor was associated with the survival rate, while funnel plots played an essential role in detecting potential publication bias.

Sensitivity and subgroup analyses were performed according to the following criteria: implant length, implant protrusion length, grafting, and smoking.

## Results

### Search process

Initially, we retrieved 1300 articles through an electronic search, and 550 duplicate articles were directly identified. Hence, the remaining 750 studies were screened based on the titles and abstracts. Case reports, systematic reviews, and studies related to animals, cadavers, models, maxillary sinus pseudocysts, and the balloon technique or hydraulics technique, were excluded during this process; ultimately, 93 studies were included in the full-text analysis. Figure 1 illustrates the explicit process for the systematic review and meta-analysis.

### Excluded articles

Among the articles in the full-text analysis, studies that were duplicate publications (*n*=8) and contained insufficient information (*n*=26) were excluded. Meanwhile, 46 articles were excluded because the implants included only conventional implants or short implants (*n*=35), the number of short implants was less than 15 (*n*=7), and no failure was reported (*n*=6). Finally, 11 articles met the inclusion criteria [19–29].

In the randomized controlled trial of Sahrmann P et al. (2016), ten short implants and fifteen conventional implants did not encounter any adverse effects during the 1-year follow-up [12]. Short implants presented a marginal bone loss of 0.16±0.62 mm, while conventional implants presented a marginal bone loss of 0.33±0.71 mm.

Shi JY et al. (2019) reported a randomized controlled trial in which no implant failed during the 1-year follow-up. Seventy short implants plus TSFE presented a marginal bone loss of 0.47 ± 0.43 mm, and 73 conventional implants plus TSFE presented a marginal bone loss of 0.52 ± 0.26 mm [30].

### Included articles

None of the included studies were randomized controlled trials, and the 11 included articles consisted of 1 cohort study and 10 cross-sectional studies. Four articles were included in the study, but no adverse events were reported. The Stranmann system, developed in Switzerland, was the most common implant system in our meta-analysis. The eleven studies included patients in a healthy condition, which excluded patients who fulfilled the following criteria: (a) smoked more than 20 cigarettes a day (b) had an uncontrolled disease, such as diabetes mellitus, osteoporosis, and active periodontitis, and (c) had experienced radiotherapy of the head and neck.

A significant proportion of patients in five studies underwent bone graft surgery, while two studies reported no bone grafts during implant surgery. In these articles, the healing period ranged from 3 to 10 months, yet the main healing time was 3 to 6 months. Four studies mentioned that a single crown was used for posterior maxillary rehabilitation, whereas a splinted crown was applied during the restoration in two articles to prevent restorative complications; five studies provided no information about the restorative method. Specific information on the studies is shown in Supplemental Table 1, and an analysis of the literature quality is shown in Supplemental Table 2. One study scored 19 points, three studies scored 18 points, three studies scored 17 points, and the other studies scored 16 points; thus, an overall rate of 17.09 points on the STROBE checklist was obtained.

### Outcome description

#### The survival rate during the healing period

**Fig. 2:**
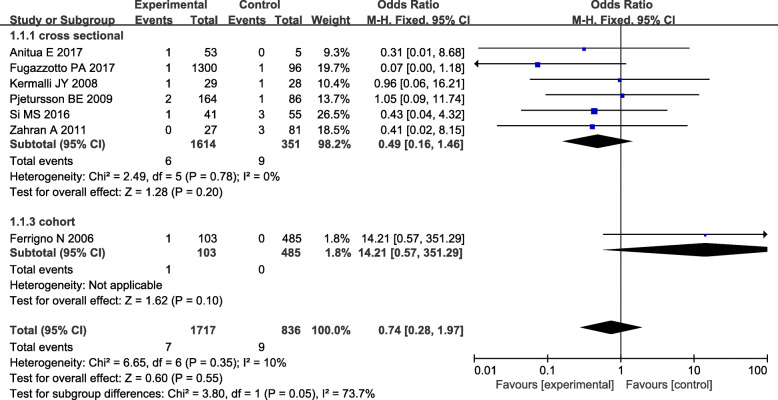
Forest plot for the survival rate during the healing period between short implants combined with TSFE (experimental group) and conventional implants combined with TSFE (control group). TSFE, transcrestal sinus floor elevation

An analysis of the 2553 implants enrolled in seven studies revealed no significant differences between the two groups (total: OR=0.74, 95% CI: 0.28-1.97, RD=−0.00, 95% CI: −0.02-0.01, cross-sectional subgroup: OR=0.49, 95% CI: 0.16-1.46, RD=−0.01, 95% CI: −0.03-0.01), indicating that implant length may not have had an effect on early failure (Fig. 2). The fixed-effects model was used on account of limited heterogeneity among the studies (*P* value=0.35, *I*^2^=10%, Fig. 2).

#### One-year survival rate

**Fig. 3:**
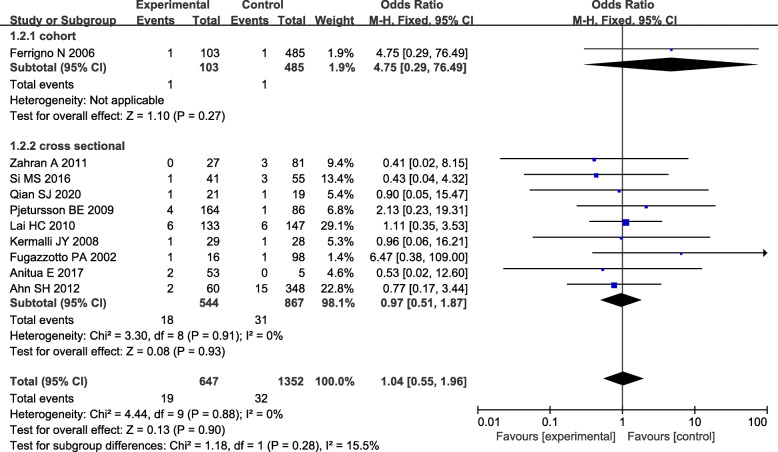
Forest plot of the 1-year survival rate between short implants combined with TSFE (experimental group) and conventional implants combined with TSFE (control group)

Ten studies included in a meta-analysis examining the 1-year survival rate after loading suggested no significant difference between the two groups (OR=1.04, 95% CI: 0.55-1.96, RD=0.00, 95% CI: −0.02-0.02, cross-sectional subgroup: OR=0.97, 95% CI: 0.51-1.87). A fixed-effects model was applied to this meta-analysis due to the lack of significant heterogeneity (*P* value=0.88, *I*^2^=0%, Fig. 3).

#### Three-year survival rate

**Fig. 4:**
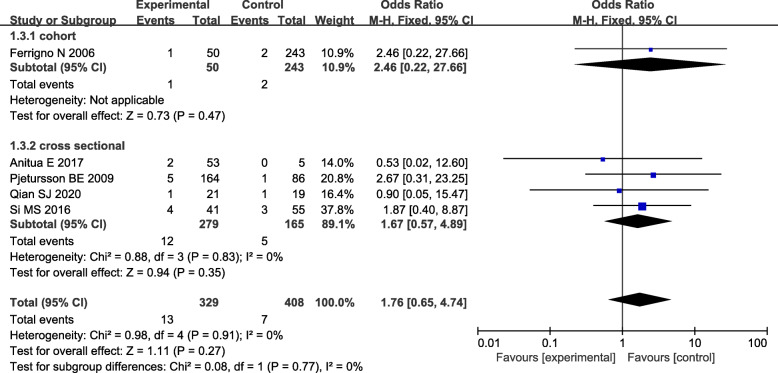
Forest plot of the 3-year survival rate between short implants combined with TSFE (experimental group) and conventional implants combined with TSFE (control group)

Five articles reported the survival rate after 3 years and demonstrated no significant difference between the two groups (*P* value=0.91, *I*^2^=0%, OR=1.76, 95% CI: 0.65-4.74, RD=0.02, 95% CI: −0.01-0.05); however, the survival rate in the experimental group (implant length ≤ 8 mm) was relatively lower than that in the control group implant length > 8 mm, Fig. 4).

#### Long-term clinical outcome

Few articles mentioned the long-term clinical outcome of short implants combined with TSFE; thus, we could not perform a meta-analysis, and the long-term clinical outcome can be described as follows.

Qian SJ et al. (2019) reported that two of 21 short implants and one of nineteen conventional implants failed during the 10-year follow-up. Therefore, the 10-year cumulative survival rate of short implants was 90.7%, and the 10-year cumulative survival rate of conventional implants was 95.0%. However, there was no significant difference between the short implants and conventional implants [28].

Fugazzotto PA. (2017) reported that the 10-year success rate of short implants was 98.8%, and the 10-year success rate of conventional implants was 100% [19].

#### Subgroup and sensitivity analysis

Subgroup and sensitivity analyses were conducted to assess various factors associated with this analysis (Supplemental Table 3). Regarding implant length (implant length ranging from 7 to 8 mm versus implant length > 8 mm**)**, no significant difference was observed between the two groups (early failure: heterogeneity: *P* value=0.48, *I*^2^=0%; OR=0.98, 95% CI: 0.33-2.88, *P* value=0.97, 1-year follow-up: heterogeneity: *P*=0.90, *I*^2^=0%; OR=0.97, 95% CI: 0.50-1.89, *P* value=0.93), and another subgroup (implant length ranging from 5 mm to 8 mm versus implant length > 8 mm) revealed the same result (early failure: heterogeneity: *P* value=0.50, *I*^2^=0%; OR=0.15, 95% CI: 0.02-1.19, *P* value=0.07, 1-year follow-up: heterogeneity: *P* value=0.81, *I*^2^=0%, OR=0.73, 95% CI: 0.08-6.31, *P* value=0.78).

When the implant protrusion length was similar between the two groups, the study revealed no significant difference between the two groups (short implants with TSFE versus conventional implants with TSFE, early failure: heterogeneity: *P* value=0.25, *I*^2^=24%; OR=0.82, 95% CI: 0.29-2.33, *P* value=0.70, 1-year follow-up: heterogeneity: *P* value=0.82, *I*^2^=0%; OR=1.22, 95% CI: 0.58-2.56, *P* value=0.59). When the implant protrusion length was different, which indicated that the residual bone height was similar, there was no significant difference between the two groups (early failure: heterogeneity: not estimated; OR=0.41, 95% CI: 0.02-8.15, *P* value=0.56, 1-year follow-up: heterogeneity: *P* value=0.71, *I*^2^=0%; OR=0.66, 95% CI: 0.17-2.53, *P*=0.55). Additional analyses are displayed in Supplemental Table 3.

### Publication bias

**Fig. 5:**
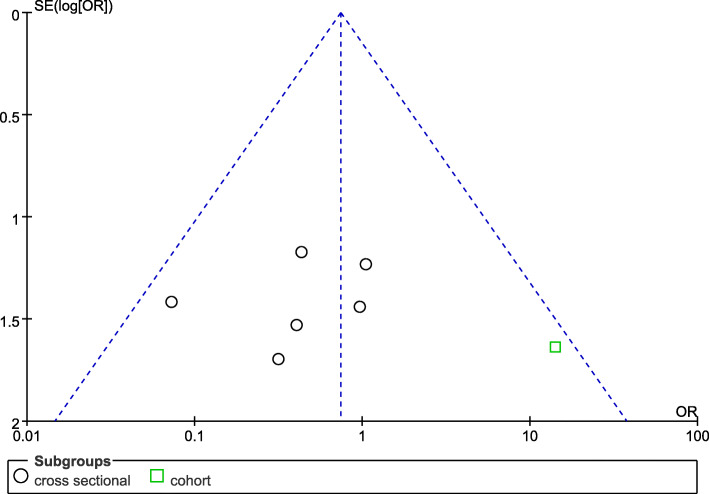
Funnel plot of the survival rate during the healing period between short implants combined with TSFE (experimental group) and conventional implants combined with TSFE (control group)

**Fig. 6:**
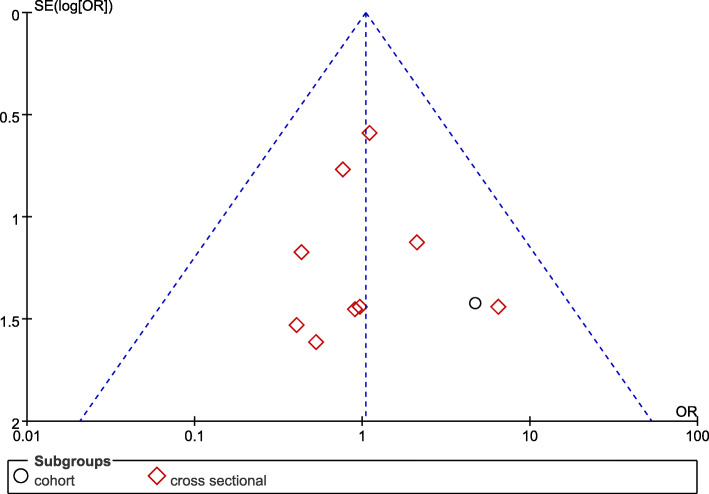
Funnel plot for the 1-year survival rate between short implants combined with TSFE (experimental group) and conventional implants combined with TSFE (control group)

As shown in two funnel plots (Figs. 5 and 6), the basic symmetry of the figures implied that there was no publication bias in the two comparative studies. However, we should be cautious about this conclusion because the number of studies was not more than 10.

## Discussion

In the present systematic review, the failure rates were assessed based on the identified studies. A similar survival rate was discovered by comparing the experimental group (length **≤** 8 mm) with the control group (implant length > 8 mm). With regard to the subgroup analysis, no significant difference between the two groups was discovered (TSFE with short implants versus TSFE with conventional implants), when the implant protrusion length was similar between the two groups. In other words, the implant length was selected mainly according to residual bone height. Nonetheless, it must be noted that all of the included articles were observational studies due to particularities in implant dentistry; therefore, there were few randomized controlled trials to confirm our results. Although a few randomized controlled studies mentioned the survival rate of conventional implants (length > 8 mm) and short implants (length≤ 8 mm) in TSFE [9–12, 30], no adverse events were reported in these studies, which gave these studies limited significance for this meta-analysis. Furthermore, TSFE with the simultaneous use of short implants (length≤ 8 mm) has been reported in several studies, but the details regarding lost implants could not be extracted from the articles; therefore, the implant survival rate could not be calculated.

There was a satisfactory survival rate of short implants combined with TSFE when the residual bone height was 4 to 8 mm in the posterior maxillary area according to network meta-analysis [31]. Nevertheless, direct comparisons were performed in our studies. The reason why we did not restrict the scope of residual bone height (RBH) is that it is still possible to implant ultrashort implants combined with crestal maxillary sinus lifting when the residual bone height is severely insufficient (< 4 mm). The major factor we controlled for was the implant protrusion length, which means that the length of the experimental group entering the maxillary sinus was similar to or less than that of the control group. Regarding the implant protrusion length, a higher penetrating length means an increased risk of Schneiderian membrane tears, which increases the likelihood of infection. However, it does not have a marked influence on the implant survival rate as long as proper measures are taken [32, 33]. Reducing the length of implant penetration can diminish the patient’s pain and trauma to some extent. In addition, conventional implants may not be suitable for pseudocysts of the maxillary sinus in some circumstances, and short implants could be applied under these circumstances [34].

However, there was a controversial debate regarding short implants due to the crown-root ratio, which may lead to more mechanical complications [35]. In recent years, comparing extrashort implants (length ≤ 6 mm) in native bone with longer implants (≥ 10 mm) after bone augmentation, similar success rates were found in both groups [12, 13, 36–38]. With improvements in implant surface modifications, short implants have been widely used in dental surgery, and various studies have focused on short implants in native bone. Whether implant length influenced the effects of TSFE was the principal purpose of our research.

One study reported that the 5-year success rate exceeded 94.6% [39], while another study obtained a success rate of 91.9% [40], which was similar to the success rates of short implants (length ≤ 8 mm) in native bone.

Nevertheless, many modified techniques ensure better outcomes of the operation, resulting in a greater length protruding into the sinus without perforation of the Schneiderian membrane [41]. Many clinicians are accustomed to using implants equal to 10 mm or more when lifting the maxillary sinus, as illustrated in the ITI (International Team for Implantology) 2018 Consensus Conference, because short implants show higher variability and lower predictability [42]. In our opinion, a short implant (length ≤ 8 mm) is an option for TSFE in severe bone loss. The treatment approach, regarding the poor available bone height in the posterior maxilla, needs to be described to the patients, and the patients can choose from among the following options:

- Short implants combined with TSFE
- Conventional implants combined with TSFE
- Lateral sinus floor elevation with simultaneous or delayed conventional implants. Of course, we have our own judgment as dentists, but we should respect the patient’s choice as well.

Few systematic reviews related to TSFE have demonstrated that failures before loading account for the main proportion of failures [4]. Early failure may originate from uncontrolled periodontal disease, endodontic disease of adjacent teeth, excessive heat production during surgery, infection, smoking, etc. Meanwhile, the thickness of the mucous membrane of the maxillary sinus was a factor influencing the possibility of perforation. Membranes that are too thick or too thin may increase the incidence of perforation, although perforation might not influence the survival rate of implants. In the present study, 11 studies including 2384 implants acquired a satisfactory survival rate during the healing time, which was not in accordance with previous studies [43–45].

### Limitation of our study

Several limitations exist in our studies: (a) an insufficient number of comparative studies may have led to erroneous conclusions; (b) the healing time and surgical instruments varied among the studies; (c) different implant systems were used; (d) no randomized trials were included; (e) limited long-term results were included; (f) there was moderate heterogeneity in the analysis of early failures; (g) the thickness of the mucous membrane of the maxillary sinus (degree of the thickening of the mucous membrane) was not known in these studies, which might influence the possibility of perforation; and (h) smoking habits were not known in several studies.

### Strength of our study

The strength of our study was as follows: we compared short implants in TSFE with conventional implants in TSFE from different follow-up times, and there were few system reviews about it.

## Conclusion

Within the restricted context of several noncontrolled studies, insufficient evidence could affirm that conventional implants with TSFE presented a higher survival rate than short implants combined with TSFE when residual bone height was poor and the protrusion length of the short implants was lower or similar to that of conventional implants, which was different from previous studies. Comparing short implants with conventional implants, similar survival rates were obtained over the short term, and long-term randomized trials are required to confirm this conclusion. Nevertheless, we must be cautious regarding this conclusion owing to the insufficient number of comparative articles that were included in this meta-analysis. In the future, long-term randomized trials are required to affirm the clinical effect of short implants combined with TSFE.

## Supplementary Information

**Additional file 1: Supplemental Table 1**. Details of the studies. **Supplemental Table 2** Quality assessment. **Supplemental Table 3** Subgroup and sensitivity analysis abbreviations: †: confidence interval ‡: odds ratio.

## Data Availability

Not applicable.

## Abbreviations

RBH: Residual bone height
TSFE: Transcrestal sinus floor elevation
