# Supplementary material for: The survival rate of transcrestal sinus floor elevation combined with short implants: a systematic review and meta-analysis of observational studies
Source: Int J Implant Dent. 2021 May 20;7:41. doi: 10.1186/s40729-021-00325-y (PMC8134646; doi:10.1186/s40729-021-00325-y)
Supplement: Supplementary file 1 — Additional file 1: Supplemental Table 1. Details of the studies. Supplemental Table 2 Quality assessment. Supplemental Table 3 Subgroup and sensitivity analysis abbreviations: †: confidence interval ‡: odds ratio. [file 40729_2021_325_MOESM1_ESM.zip › support material(excluded articles).docx]

**Excluded articles:**

**No failure reported:**

Diserens V, Mericske E, Mericske-Stern R. Radiographic analysis of the transcrestal sinus floor elevation: short-term observations. *Clin Implant Dent Relat Res.* 2005;7:70–78.

Zheng X, Teng M, Zhou F, Ye J, Li G, Mo A. Influence of Maxillary Sinus Width on Transcrestal Sinus Augmentation Outcomes: Radiographic Evaluation Based on Cone Beam CT. *Clin Implant Dent Relat Res.* 2016;18:292–300.

Checchi L, Felice P, Antonini ES, Cosci F, Pellegrino G, Esposito M. Crestal sinus lift for implant rehabilitation: a randomised clinical trial comparing the Cosci and the Summers techniques. A preliminary report on complications and patient preference. *Eur J Oral Implantol*. 2010;3:221–232.

Taschieri S, Corbella S, Del Fabbro M. Mini-invasive osteotome sinus floor elevation in partially edentulous atrophic maxilla using reduced length dental implants: interim results of a prospective study. *Clin Implant Dent Relat Res.* 2014;16:185–193.

Sahrmann P, Naenni N, Jung RE, et al. Success of 6-mm Implants with Single-Tooth Restorations: A 3-year Randomized Controlled Clinical Trial. *J Dent Res*. 2016;95:623‐628.

Shi JY, Li Y, Qiao SC, Gu YX, Xiong YY, Lai HC. Short versus longer implants with osteotome sinus floor elevation for moderately atrophic posterior maxillae: A 1-year randomized clinical trial. *J Clin Periodontol*. 2019;46(8):855‐862.

**Only short implants reported:**

Nizam N, Gürlek Ö, Kaval ME. Extra-Short Implants with Osteotome Sinus Floor Elevation: A Prospective Clinical Study. *Int J Oral Maxillofac Implants.* 2020;35:415–422.

Yang J, Xia T, Fang J, Shi B. Radiological Changes Associated with New Bone Formation Following Osteotome Sinus Floor Elevation (OSFE): A Retrospective Study of 40 Patients with 18-Month Follow-Up. *Med Sci Monit.* 2018;24:4641–4648.

Qian SJ, Gu YX, Mo JJ, Qiao SC, Zhuang LF, Lai HC. Resonance frequency analysis of implants placed with osteotome sinus floor elevation in posterior maxillae. *Clin Oral Implants* Res. 2016;27:113–119.

Deporter DA, Caudry S, Kermalli J, Adegbembo A. Further data on the predictability of the indirect sinus elevation procedure used with short, sintered, porous-surfaced dental implants. *Int J Periodontics Restorative Dent.* 2005;25:585–593.

Gu YX, Shi JY, Zhuang LF, Qian SJ, Mo JJ, Lai HC. Transalveolar sinus floor elevation using osteotomes without grafting in severely atrophic maxilla: a 5-year prospective study. *Clin Oral Implants Res.* 2016;27:120–125.

Ng P, Hu X, Wan S, Mo H, Deng F. Clinical Outcomes of Bicortical Engagement Implants in Atrophic Posterior Maxillae: A Retrospective Study with 1 to 5 Years Follow-up. *Int J Periodontics Restorative Dent*. 2018;38:e96–e104.

Yu H, Wang X, Qiu L. Outcomes of 6.5-mm Hydrophilic Implants and Long Implants Placed with Lateral Sinus Floor Elevation in the Atrophic Posterior Maxilla: A Prospective, Randomized Controlled Clinical Comparison. *Clin Implant Dent Relat Res.* 2017;19:111–122.

Nedir R, Nurdin N, Abi Najm S, El Hage M, Bischof M. Short implants placed with or without grafting into atrophic sinuses: the 5-year results of a prospective randomized controlled study. *Clin Oral Implants Res.* 2017;28:877–886.

Qin L, Lin SX, Guo ZZ, et al. Influences of Schneiderian membrane conditions on the early outcomes of osteotome sinus floor elevation technique: a prospective cohort study in the healing period. *Clin Oral Implants Res.* 2017;28:1074–1081.

Schmidlin PR, Müller J, Bindl A, et al. Sinus floor elevation using an osteotome technique without grafting materials or membranes. *Int J Periodontics Restorative Dent.* 2008;28:401–409.

Santagata M, Guariniello L, D'amato S, Tozzi U, Rauso R, Tartaro G. Augmentation of atrophic posterior maxilla by short implants and osteotome technique. *Stomatologija.* 2012;14:85–88.

**Repeated publication:**

Anitua E, Flores J, Alkhraisat MH. Transcrestal Sinus Lift Using Platelet Concentrates in Association to Short Implant Placement: A Retrospective Study of Augmented Bone Height Remodeling. *Clin Implant Dent Relat Res.* 2016;18:993–1002.

Deporter D, Todescan R, Caudry S. Simplifying management of the posterior maxilla using short, porous-surfaced dental implants and simultaneous indirect sinus elevation. *Int J Periodontics Restorative Dent.* 2000;20:476–485.

Anitua E, Flores J, Alkhraisat MH. Transcrestal Sinus Floor Augmentation by Sequential Drilling and the Use of Plasma Rich in Growth Factors. Int J Oral Maxillofac Implants. 2017;32:e167–e173.

Zhang XM, Shi JY, Gu YX, Qiao SC, Mo JJ, Lai HC. Clinical Investigation and Patient Satisfaction of Short Implants Versus Longer Implants with Osteotome Sinus Floor Elevation in Atrophic Posterior Maxillae: A Pilot Randomized Trial. *Clin Implant Dent Relat Res.* 2017;19:161–166.

Taschieri S, Del Fabbro M. Postextraction osteotome sinus floor elevation technique using plasma-rich growth factors. *Implant Dent.* 2011;20:418–424.

Nedir R, Nurdin N, Khoury P, Bischof M. Short Implants Placed with or without Grafting in Atrophic Sinuses: The 3-Year Results of a Prospective Randomized Controlled Study. *Clin Implant Dent Relat Res.* 2016;18:10–18.

Nedir R, Bischof M, Vazquez L, Nurdin N, Szmukler-Moncler S, Bernard JP. Osteotome sinus floor elevation technique without grafting material: 3-year results of a prospective pilot study. *Clin Oral Implants Res.* 2009;20:701–707.

Nedir R, Nurdin N, Khoury P, et al. Osteotome sinus floor elevation with and without grafting material in the severely atrophic maxilla. A 1-year prospective randomized controlled study. *Clin Oral Implants Res.* 2013;24:1257–1264.

**Insufficient information:**

French D, Nadji N, Shariati B, Hatzimanolakis P, Larjava H. Survival and Success Rates of Dental Implants Placed Using Osteotome Sinus Floor Elevation Without Added Bone Grafting: A Retrospective Study with a Follow-up of up to 10 Years. *Int J Periodontics Restorative Dent.* 2016;36:s89–s97.

Uckan S, Tamer Y, Deniz K. Survival rates of implants inserted in the maxillary sinus area by internal or external approach. *Implant Dent.* 2011;20:476–479.

Trombelli L, Franceschetti G, Rizzi A, Minenna P, Minenna L, Farina R. Minimally invasive transcrestal sinus floor elevation with graft biomaterials. A randomized clinical trial. *Clin Oral Implants Res.* 2012;23:424–432.

Rammelsberg P, Mahabadi J, Eiffler C, Koob A, Kappel S, Gabbert O. Radiographic monitoring of changes in bone height after implant placement in combination with an internal sinus lift without graft material. *Clin Implant Dent Relat Res.* 2015;17:e267–e274.

Perelli M, Abundo R, Corrente G, Saccone C. Short (5 and 7 mm long) porous implants in the posterior atrophic maxilla: a 5-year report of a prospective single-cohort study. *Eur J Oral Implantol*. 2012;5:265–272.

Liu H, Liu R, Wang M, Yang J. Immediate implant placement combined with maxillary sinus floor elevation utilizing the transalveolar approach and nonsubmerged healing for failing teeth in the maxillary molar area: A randomized controlled trial clinical study with one-year follow-up. *Clin Implant Dent Relat Res.* 2019;21:462–472.

Verdugo F, Uribarri A, Laksmana T, D'addona A. Long-term stable vertical bone regeneration after sinus floor elevation and simultaneous implant placement with and without grafting. *Clin Implant Dent Relat Res.* 2017;19:1054–1060.

Rizzo R, Quaranta A, De Paoli M, Rappelli G, Piemontese M. Three-Dimensional Bone Augmentation and Immediate Implant Placement via Transcrestal Sinus Lift:8-Year Clinical Outcomes. *Int J Periodontics Restorative Dent.* 2018;38:423-429.

Rawat A, Thukral H, Jose A. Indirect Sinus Floor Elevation Technique with Simultaneous Implant Placement without Using Bone Grafts. *Ann Maxillofac Surg*. 2019;9:96–102.

Lindgren C, Mordenfeld A, Hallman M. A prospective 1-year clinical and radiographic study of implants placed after maxillary sinus floor augmentation with synthetic biphasic calcium phosphate or deproteinized bovine bone. *Clin Implant Dent Relat Res.* 2012;14:41–50.

Diss A, Dohan DM, Mouhyi J, Mahler P. Osteotome sinus floor elevation using Choukroun's platelet-rich fibrin as grafting material: a 1-year prospective pilot study with microthreaded implants. *Oral Surg Oral Med Oral Pathol Oral Radiol Endod.* 2008;105:572–579.

Brägger U, Gerber C, Joss A, et al. Patterns of tissue remodeling after placement of ITI dental implants using an osteotome technique: a longitudinal radiographic case cohort study. *Clin Oral Implants Res.* 2004;15:158–166.

Lo Giudice G, Iannello G, Terranova A, Lo Giudice R, Pantaleo G, Cicciù M. Transcrestal Sinus Lift Procedure Approaching Atrophic Maxillary Ridge: A 60-Month Clinical and Radiological Follow-Up Evaluation. *Int J Dent.* 2015;2015:261652.

Abdulkarim HH, Miley DD, McLeod DE, Garcia MN. Short-term evaluation of bioactive glass using the modified osteotome sinus elevation technique. *Implant Dent.* 2013;22:491–498.

Browaeys H, Defrancq J, Dierens MC, et al. A retrospective analysis of early and immediately loaded osseotite implants in cross-arch rehabilitations in edentulous maxillas and mandibles up to 7 years. *Clin Implant Dent Relat Res.* 2013;15:380–389.

Bruckmoser E, Gruber R, Steinmassl O, et al. Crestal Sinus Floor Augmentation Using Hydraulic Pressure and Vibrations: A Retrospective Single Cohort Study. *Int J Oral Maxillofac Implants.* 2018;33:1149–1154.

Chandra RV, Suvvari N, Reddy AA. Trephine Core Procedure Versus Bone-Added Osteotome Sinus Floor Elevation in the Augmentation of the Sinus Floor: A Comparative Clinical and Radiographic Study.*Int J Oral Maxillofac Implants.* 2018;33:425–432.

Franceschetti G, Farina R, Minenna L, et al. The impact of graft remodeling on peri-implant bone support at implants placed concomitantly with transcrestal sinus floor elevation: A multicenter, retrospective case series. *Clin Oral Implants Res.* 2020;31:105–120.

Franceschetti G, Farina R, Stacchi C, Di Lenarda R, Di Raimondo R, Trombelli L. Radiographic outcomes of transcrestal sinus floor elevation performed with a minimally invasive technique in smoker and non-smoker patients. *Clin Oral Implants Res.* 2014;25:493–499.

Garbacea A, Lozada JL, Church CA, et al. The incidence of maxillary sinus membrane perforation during endoscopically assessed crestal sinus floor elevation: a pilot study. *J Oral Implantol*. 2012;38:345–359.

Hsu A, Seong WJ, Wolff R, et al. Comparison of Initial Implant Stability of Implants Placed Using Bicortical Fixation, Indirect Sinus Elevation, and Unicortical Fixation. *Int J Oral Maxillofac Implants.* 2016;31:459–468.

Bruschi GB, Scipioni A, Calesini G, Bruschi E. Localized management of sinus floor with simultaneous implant placement: a clinical report. *Int J Oral Maxillofac Implants.* 1998;13:219–226.

Komarnyckyj OG, London RM. Osteotome single-stage dental implant placement with and without sinus elevation: a clinical report.*Int J Oral Maxillofac Implants.* 1998;13:799–804.

Spinato S, Bernardello F, Galindo-Moreno P, Zaffe D. Maxillary sinus augmentation by crestal access: a retrospective study on cavity size and outcome correlation. *Clin Oral Implants Res.* 2015;26:1375–1382.

Strietzel FP, Nowak M, Küchler I, Friedmann A. Peri-implant alveolar bone loss with respect to bone quality after use of the osteotome technique: results of a retrospective study. *Clin Oral Implants Res.* 2002;13:508–513.

Tetsch J, Tetsch P, Lysek DA. Long-term results after lateral and osteotome technique sinus floor elevation: a retrospective analysis of 2190 implants over a time period of 15 years. *Clin Oral Implants Res.* 2010;21:497–503.

**implant length≥9mm:**

Sesma N, Pannuti C, Cardaropoli G. Retrospective clinical study of 988 dual acid-etched implants placed in grafted and native bone for single-tooth replacement. *Int J Oral Maxillofac Implants.* 2012;27:1243-1248.

Mahesh L, Agarwal A, Guirado JC, Bali P, Poonia N. Survival of Implants after Indirect Maxillary Sinus Elevation Procedure: A Two Years Longitudinal Study. *J Contemp Dent Pract.* 2019 ;20:504-507.

Rammelsberg P, Kilian S, Büsch C, Kappel S. The effect of transcrestal sinus-floor elevation without graft on the long-term prognosis of maxillary implants. *J Clin Periodontol.* 2020.

Bernardello F, Felice P, Spinato S, et al. Stage Characterization and Marginal Bone Loss Evaluation Up to 96 Months of Crestal Sinus Augmentation With Sequential Drills: A Retrospective Study. *Implant Dent.* 2015;24:642-649.

Bernardello F, Righi D, Cosci F, Bozzoli P, Soardi CM, Spinato S. Crestal sinus lift with sequential drills and simultaneous implant placement in sites with <5 mm of native bone: a multicenter retrospective study. *Implant Dent.* 2011;20:439-444.

AlGhamdi AS. Osteotome maxillary sinus lift using bovine bone and calcium

sulfate: a case series. *Clin Implant Dent Relat Res.* 2013;15:153-159.

Baldi D, Menini M, Pera F, Ravera G, Pera P. Sinus floor elevation using osteotomes or piezoelectric surgery. *Int J Oral Maxillofac Surg.* 2011;40:497-503.

Bruschi GB, Crespi R, Capparè P, Gherlone E Transcrestal sinus floor elevation: a retrospective study of 46 patients up to 16 years. *Clin Implant Dent Relat Res.* 2012;14:759-767.

Bruschi GB, Crespi R, Capparè P, Bravi F, Bruschi E, Gherlone E. Localized management of sinus floor technique for implant placement in fresh molar sockets. *Clin Implant Dent Relat Res.* 2013;15:243-250.

Caban J, Fermergård R, Abtahi J. Long-term evaluation of osteotome sinus floor elevation and simultaneous placement of implants without bone grafts: 10-Year radiographic and clinical follow-up. *Clin Implant Dent Relat Res.* 2017;19:1023-1033.

Chen HH, Lin YC, Lee SY, Chang LY, Chen BJ, Lai YL. Influence of Sinus Floor Configuration on Grafted Bone Remodeling After Osteotome Sinus Floor Elevation. *J Periodontol.* 2017;88:10-16

Nahlieli O. Dynamic implant valve approach for dental implant procedures. *Chin J Dent Res.* 2014;17:15-21.

Crespi R, Capparè P, Gherlone E. Osteotome sinus floor elevation and simultaneous implant placement in grafted biomaterial sockets: 3 years of follow-up. *J Periodontol.* 2010;81:344-349.

El Hage M, Nurdin N, Abi Najm S, Bischof M, Nedir R. Osteotome Sinus Floor Elevation Without Grafting: A 10-Year Study of Cone Beam Computerized Tomography vs Periapical Radiography. *Int J Periodontics Restorative Dent*. 2019;39:e89–e97.

Jesch P, Bruckmoser E, Bayerle A, Eder K, Bayerle-Eder M, Watzinger F. A pilot-study of a minimally invasive technique to elevate the sinus floor membrane and place graft for augmentation using high hydraulic pressure: 18-month follow-up of 20 cases. *Oral Surg Oral Med Oral Pathol Oral Radiol.* 2013;116:293-300.

Urban IA, Lozada JL. A prospective study of implants placed in augmented sinuses with minimal and moderate residual crestal bone: results after 1 to 5 years. *Int J Oral Maxillofac Implants.* 2010;25:1203-1212.

Kim SM, Park JW, Suh JY, Sohn DS, Lee JM. Bone-added osteotome technique versus lateral approach for sinus floor elevation: a comparative radiographic study. *Implant Dent.* 2011;20:465-470.

Kolerman R, Moses O, Artzi Z, Barnea E, Tal H. Maxillary sinus augmentation by the crestal core elevation technique. *J Periodontol.* 2011;82:41-51.

Nishida T, Takenouchi Y, Mori K, Ariji M, Nishida K, Ito K.. Remodeling of autogenous bone grafts after osteotome sinus floor elevation assessed by limited cone beam computed tomography. *Int J Dent.* 2013;2013:931708.

Schleier P, Bierfreund G, Schultze-Mosgau S, Moldenhauer F, Küpper H, Freilich M. Simultaneous dental implant placement and endoscope-guided internal sinus floor elevation: 2-year post-loading outcomes. *Clin Oral Implants Res.* 2008;19:1163-1170.

Spinelli D, DE Vico G, Condò R, Ottria L, Arcuri C. Transcrestal guided sinus lift without grafting materials: a 36 months clinical prospective study. *Oral Implantol (Rome).* 2016;8:74–86.

Thor A, Wannfors K, Sennerby L, Rasmusson L. Reconstruction of the severely resorbed maxilla with autogenous bone, platelet-rich plasma, and implants: 1-year results of a controlled prospective 5-year study. *Clin Implant Dent Relat Res.* 2005;7:209–220.

Volpe S, Lanza M, Verrocchi D, Sennerby L. Clinical outcomes of an osteotome technique and simultaneous placement of Neoss implants in the posterior maxilla. *Clin Implant Dent Relat Res.* 2013;15:22–28.

Zitzmann NU, Schiirer. Sinus elevation procedures in the resorbed posterior maxilla. *Oral And Maxillofacial Surgery.* 1998;85:8-17

**The number of short implant <5:**

Park WB, Kim YJ, Kang KL, Lim HC, Han JY. Long-term outcomes of the implants accidentally protruding into nasal cavity extended to posterior maxilla due to inferior meatus pneumatization. *Clin Implant Dent Relat Res.* 2020;22:105-111

Fornell J, Johansson LÅ, Bolin A, Isaksson S, Sennerby L. Flapless, CBCT-guided osteotome sinus floor elevation with simultaneous implant installation. I: radiographic examination and surgical technique. A prospective 1-year follow-up. *Clin Oral Implants Res.* 2012;23:28-34.

Gabbert O, Koob A, Schmitter M, Rammelsberg P. Implants placed in combination with an internal sinus lift without graft material: an analysis of short-term failure. *J Clin Periodontol.* 2009;36:177-183.

Bae OY, Kim YS, Shin SY, Kim WK, Lee YK, Kim SH. Clinical Outcomes of Reamer- vs Osteotome-Mediated Sinus Floor Elevation with Simultaneous Implant Placement: A 2-Year Retrospective Study. *Int J Oral Maxillofac Implants.* 2015;30:925–930.

Calasans-Maia MD, Mourão CF, Alves AT, Sartoretto SC, de Uzeda MJ, Granjeiro JM. Maxillary Sinus Augmentation with a New Xenograft: A Randomized Controlled Clinical Trial. *Clin Implant Dent Relat Res.* 2015;17:e586–e593.

Zhang Q, Zhang LL, Yang Y, Lin YZ, Miron RJ, Zhang YF. Improvement of Implant Placement after Bone Augmentation of Severely Resorbed Maxillary Sinuses with 'Tent-Pole' Grafting Technique in Combination with rhBMP-2. *Chin J Dent Res.* 2017;20:9–17.

Franceschetti G, Trombelli L, Minenna L, Franceschetti G, Farina R. Learning Curve of a Minimally Invasive Technique for Transcrestal Sinus Floor Elevation: A Split-Group Analysis in a Prospective Case Series With Multiple Clinicians. *Implant Dent.* 2015;24:517–526.
