# Supplementary material for: The survival rate of transcrestal sinus floor elevation combined with short implants: a systematic review and meta-analysis of observational studies
Source: Int J Implant Dent. 2021 May 20;7:41. doi: 10.1186/s40729-021-00325-y (PMC8134646; doi:10.1186/s40729-021-00325-y)
Supplement: Supplementary file 1 — Additional file 1: Supplemental Table 1. Details of the studies. Supplemental Table 2 Quality assessment. Supplemental Table 3 Subgroup and sensitivity analysis abbreviations: †: confidence interval ‡: odds ratio. [file 40729_2021_325_MOESM1_ESM.zip › table.docx]

| Supplemental Table 1. Details of the studies | | | | | | | | | |
| --- | --- | --- | --- | --- | --- | --- | --- | --- | --- |
| No | **Author** | **Study design** | **Country** | **No. of**  **patients** | **No.of**  **implants** | **System** | **Graft** | **Residual bone height** | **Follow-up** |
| 1 | Paul A. Fugazzotto | Cross-sectional | USA | 1305 | 1344 | Straumann | unknown | 4-6 | 20 year |
| 2 | Paul A. Fugazzotto | Cross-sectional | USA | 103 | 116 | Straumann | unknown | unknown | 4 year |
| 3 | Eduardo Anitua | Cross-sectional | Spain | 38 | 58 | BTI | 26%graft | 4.6±1.2 | 10year |
| 4 | Zahran A | Cross-sectional | UK | 64 | 108 | Osteocare | NO | Minimum5 | 1year |
| 5 | Pjetursson.B.E | Cross-sectional | Switzerland | 181 | 250 | Straumann | 65%graft | 7.5±2.2 | 3.2year |
| 6 | Ferrigno N | cohort | Italy | 323 | 588 | Straumann | unknown | 6-9 | 1year annual |
| 7 | Hong-Chang Lai | Cross-sectional | China | 202 | 280 | Straumann | 32% graft | 2 to 8 | 1year annual |
| 8 | Sang-Hoon Ahn | Cross-sectional | Korea | 380 | 391 | unknown | unknown | 5.8±2.9 | 28.2month |
| 9 | Mi-si Si | Cross-sectional | China | 80 | 96 | Straumann | NO | 1.30-10.02 | 1-2year annual |
| 10 | Qian SJ | Cross-sectional | China | 45 | 45 | Straumann | 50% graft | 2-8 | 10 year |
| 11 | Jaffer Y. Kermalli | Cross-sectional | Canada | 25 | 57 | unknown | 98%graft | 4 to 12 | 2 year |

| Supplemental Table 2 Quality assessment | | | | | | | |
| --- | --- | --- | --- | --- | --- | --- | --- |
| Study | **item** | | | | | | |
|  | **Title and abstract** | **Introduction** | **Methods** | **Results** | **Discussion** | **Other** | **Total** |
| Fugazzotto PA. 2017 | 1 | 2 | 7 | 4 | 2 | 0 | 16 |
| Fugazzotto PA. 2002 | 1 | 2 | 7 | 4 | 2 | 0 | 16 |
| Anitua E et al. 2017 | 1 | 2 | 7 | 4 | 2 | 0 | 16 |
| Zahran A et al. 2011 | 1 | 2 | 7 | 4 | 2 | 0 | 16 |
| Pjetursson BE et al. 2009 | 1 | 2 | 7 | 4 | 3 | 0 | 17 |
| Ferrigno N et al. 2006 | 1 | 2 | 8 | 5 | 3 | 0 | 19 |
| Lai HC et al. 2010 | 1 | 2 | 7 | 4 | 3 | 1 | 18 |
| Ahn SH et al. 2012 | 1 | 2 | 7 | 4 | 3 | 0 | 17 |
| Si MS et al. 2016 | 1 | 2 | 7 | 4 | 3 | 1 | 18 |
| Kermalli JY et al. 2008 | 1 | 2 | 7 | 4 | 2 | 1 | 17 |
| Qian SJ et al. 2020 | 1 | 2 | 7 | 4 | 3 | 1 | 18 |

| Supplemental Table 3 Subgroup and sensitivity analysis abbreviations: †: confidence interval ‡: odds ratio | | | | | | | | | | | | | |
| --- | --- | --- | --- | --- | --- | --- | --- | --- | --- | --- | --- | --- | --- |
|  | **Early failure** | | | | | | **1-year** | | | | | | |
|  |  | **Heterogeneity** | | **Statistical analysis** | | |  | **Heterogeneity** | | | **Statistical analysis** | | |
|  | **n** | **P-value** | **I^2^** | **95%CI**_†_ | **OR**_‡_ | **P-value** | **n** | **P-value** | | **I^2^** | **95%CI** | **OR** | **P-value** |
| Containing implants  (length≤6mm) | 2 | 0.50 | 0% | 0.02-1.19 | 0.15 | 0.07 | 2 | 0.81 | 0% | | 0.08-6.31 | 0.73 | 0.78 |
| Mainly 7mm and 8 mm | 5 | 0.48 | 0% | 0.33-2.88 | 0.98 | 0.97 | 8 | 0.90 | | 0% | 0.50-1.89 | 0.97 | 0.93 |
| Various implant protruding length | 1 | Not estimated | | 0.02-8.15 | 0.41 | 0.56 | 2 | 0.71 | | 0% | 0.17-2.53 | 0.66 | 0.55 |
| Similar Implant protruding length | 6 | 0.25 | 24% | 0.29-2.33 | 0.82 | 0.70 | 8 | 0.82 | | 0% | 0.58-2.56 | 1.22 | 0.59 |
| Non-grafting | 2 | 0.97 | 0% | 0.07-2.63 | 0.42 | 0.36 | 2 | 0.97 | | 0% | 0.07-2.63 | 0.42 | 0.36 |
| Containing  grafting | 3 | 0.83 | 0% | 0.16-4.14 | 0.82 | 0.81 | 5 | 0.97 | | 0% | 0.49-2.76 | 1.16 | 0.74 |
| Straumann | 4 | 0.10 | 53% | 0.26-2.85 | 0.87 | 0.81 | 6 | 0.66 | | 0% | 0.59-2.86 | 1.30 | 0.51 |
| No smoker | 2 | 0.11 | 61% | 0.31-8.78 | 1.65 | 0.55 | 2 | 0.23 | | 32% | 0.18-7.02 | 1.13 | 0.90 |
| Containing smokers | 4 | 0.48 | 0% | 0.15-1.86 | 0.53 | 0.32 | 6 | 0.78 | | 0% | 0.53-2.57 | 1.17 | 0.70 |
